# Patient-Reported Outcomes After MCAT with Connective Tissue Graft: A Retrospective Pooled Analysis of Three Split-Mouth Randomized Controlled Trials

**DOI:** 10.3390/healthcare14111598

**Published:** 2026-06-05

**Authors:** Nela Molga-Chlipała, Małgorzata Wyszyńska-Pomian, Bartłomiej Górski

**Affiliations:** Department of Periodontology and Oral Diseases, Medical University of Warsaw, 02-091 Warsaw, Poland; malgorzata.wyszynska-pomian@wum.edu.pl (M.W.-P.); bartlomiej.gorski@wum.edu.pl (B.G.)

**Keywords:** gingival recession, patient-reported outcome measures, MCAT, subepithelial connective tissue graft

## Abstract

**Highlights:**

**What are the main findings?**

- MCAT combined with SCTG achieved predictable and comparable root coverage at 12 months, regardless of the adjunctive biomaterial used.
- Cross-linked hyaluronic acid was associated with a slightly greater clinical attachment level gain compared with other adjunctive approaches.

**What are the implications of the main findings?**

- Patient satisfaction and willingness to recommend the procedure were high across all treatment modalities.
- Integrating PROMs with clinician-reported and esthetic outcomes provides a more comprehensive and patient-centered evaluation of periodontal plastic surgery.

**Abstract:**

**Background**: Patient-reported outcome measures (PROMs) are essential for assessing postoperative morbidity and patient-centered treatment value after periodontal plastic surgery. This study evaluated PROMs following the modified coronally advanced tunnel (MCAT) combined with subepithelial connective tissue graft (SCTG) and different adjunctive biomaterials. **Methods**: This retrospective pooled secondary analysis included 64 patients treated for 275 gingival recessions using MCAT + SCTG with physiological saline (control), 24% EDTA, enamel matrix derivative (EMD), or cross-linked hyaluronic acid (HA). PROMs were the primary outcomes and included postoperative pain and swelling assessed using a 0–100 mm visual analogue scale (VAS), together with binary symptom measures recorded on days 1, 2, 4, 7, and 14. Patient satisfaction, esthetic perception, and willingness to recommend treatment were also assessed. Clinical and esthetic outcomes at 12 months served as reference parameters. **Results**: Early postoperative pain differed significantly between groups on days 1, 2, and 7 (*p* = 0.016, 0.024, and 0.033, respectively). Edema also varied significantly on days 2, 4, and 14 (*p* = 0.011, 0.001, and 0.001, respectively). The HA group showed lower pain scores on days 2 and 4, whereas EDTA was associated with greater early discomfort. Despite these differences, long-term satisfaction, esthetic perception, willingness to recommend treatment, and root coverage outcomes were high and comparable among groups (*p* > 0.05). **Conclusions**: MCAT + SCTG provides predictable and stable outcomes for multiple gingival recessions, regardless of adjunctive biomaterial. However, PROMs revealed differences in early postoperative morbidity, supporting their integration into clinical decision-making alongside clinician-reported outcomes.

## 1. Introduction

Gingival recession (GR) refers to the apical migration of gingival tissue, exposing the root surface beyond the cementoenamel junction (CEJ). Recent studies have shown that two-thirds of the global population is affected by gingival recession, and its prevalence increases with age [1,2]. Gingival recession is a multifactorial condition associated with mechanical, inflammatory, and anatomical factors [3,4]. Some studies point to gingival inflammation as the main factor in the development of gingival recession (REC) [2], but the etiology is usually multifactorial, and the appearance is attributed to multiple interacting factors [5].

For clinical decision-making and outcome assessment, gingival recessions are commonly classified according to internationally accepted systems. The most widely used contemporary classification is the Cairo classification, which categorizes gingival recessions into three types (RT1, RT2, and RT3) based on the level of interproximal clinical attachment relative to the buccal attachment loss. The Cairo classification is currently considered the standard system for the clinical assessment and prognostic evaluation of gingival recessions. RT1 defects present with no interproximal attachment loss, RT2 defects exhibit interproximal attachment loss equal to or less than the buccal loss, and RT3 defects show greater interproximal attachment loss than buccal loss. This classification provides a prognostic framework for root coverage procedures and facilitates standardized reporting and comparison of clinical outcomes across studies [6].

Contemporary patients have high esthetic expectations; therefore, gingival recession treatment remains clinically challenging [6]. By treating gingival recessions, it is possible to avoid dentin hypersensitivity, predisposition to root caries, and even tooth loss [7,8].

Given that GR therapy is frequently undertaken for esthetic reasons and symptom relief, treatment success should be evaluated not only by clinician-assessed root coverage but also by outcomes perceived by patients [9,10].

The modified coronally advanced tunnel (MCAT) combined with a subepithelial connective tissue graft (SCTG) is a well-established approach for treating multiple gingival recessions, providing predictable root coverage and favorable esthetic outcomes [11,12,13,14]. Its minimally invasive design preserves the vascular supply, although the palatal donor site may contribute to postoperative morbidity, underscoring the need for adjunctive strategies to improve healing and patient comfort.

Biologically driven adjuncts have been introduced to optimize the wound environment and soft-tissue integration, with potential implications for both clinical outcomes and patient-experienced recovery [15,16,17,18,19,20]. These agents differ in their mechanisms of action and clinical applicability. Cross-linked HA exhibits anti-inflammatory and pro-angiogenic properties and may enhance early soft-tissue healing and reduce postoperative discomfort, particularly at palatal donor sites, although its impact on long-term clinical outcomes remains unclear [20,21,22]. EMD is used to promote periodontal regeneration and may improve clinical attachment; however, its additional benefit in conjunction with connective tissue grafting is still debated and may be influenced by cost and technique sensitivity [23]. EDTA is applied for root surface conditioning to facilitate connective tissue attachment, but evidence regarding its added clinical and patient-related benefits is inconsistent [24].

Although the biological rationale for hyaluronic acid—including its anti-inflammatory and pro-angiogenic properties—is well established [20,21,24], its translation into consistent clinical benefit, particularly in terms of root coverage outcomes, has not been clearly demonstrated. Moreover, most available studies focus predominantly on clinician-reported parameters, with insufficient integration of patient-reported outcomes. Consequently, the clinical relevance of adjunctive biomaterials in MCAT procedures, especially regarding their impact on early postoperative morbidity and patient experience, remains insufficiently defined.

Patient-reported outcome measures (PROMs) capture domains directly relevant to individuals undergoing periodontal plastic surgery, including postoperative pain and swelling, functional limitations, and satisfaction with esthetic integration, thereby complementing objective clinical indices [9,10].

Therefore, in the present study, PROMs were specifically included to quantify differences in early postoperative pain and swelling between adjunctive protocols that vary in biomodification and material properties [21].

Although the clinical outcomes of the original split-mouth randomized clinical trials have been reported previously, patient-reported outcomes have not yet been evaluated in an integrated manner across the full dataset. Therefore, in the present study, PROMs were specifically included to quantify differences in early postoperative pain and swelling between adjunctive protocols that vary in biomodification and material properties [21].

Therefore, the primary objective of this pooled secondary analysis was to evaluate patient-reported outcome measures (PROMs), including postoperative morbidity trajectories and patient-perceived esthetic outcomes, following MCAT combined with SCTG with different adjunctive biomaterials. Secondary objectives included the assessment of clinical and professional esthetic outcomes as contextual reference parameters to facilitate interpretation of patient-centered results. Clinical and professional esthetic parameters were included only as contextual reference variables to facilitate the interpretation of patient-centered outcomes.

## 2. Materials and Methods

### 2.1. Study Design

The present study is a retrospective pooled secondary analysis of prospectively collected data from three independent split-mouth randomized clinical trials. In each original trial, one quadrant received the adjunctive biomaterial (EMD, EDTA, or HA), while the contralateral quadrant served as saline control. This review included 64 consecutively treated patients with 275 gingival recessions. In 20 participants, root surfaces were conditioned with 24% EDTA followed by EMD, while in the remaining 20 they were treated with 24% EDTA alone. In the hyaluronic acid group, 24 patients received application of HA directly on the root surface. The study was conducted in accordance with the Declaration of Helsinki (1975), including the amendments adopted in Tokyo in 2004, and received approval from the Bioethics Committee of the Medical University of Warsaw (KB/208/2017; KB/119/2021) and registered with ClinicalTrials.gov (NCT03354104; NCT05045586). Participants were enrolled from patients who had been referred to the Department of Periodontology and Oral Mucosa Diseases at the Medical University of Warsaw between January 2018 and April 2020 for EDTA and EMD group, and April 2021-May 2022 for HA. A single examiner (NMC) qualified patients with multiple gingival recessions for inclusion in the study, and all participants provided written informed consent. They received instruction in the roll-toothbrushing method, along with professional prophylaxis and polishing. In each case, gingival recessions in one quadrant were managed using SCTG combined with the MCAT technique.

All participants were monitored for 12 months.

### 2.2. Patient Population

A patient was qualified for the study if the following criteria were met: (1) at least two adjacent gingival recessions of type RT1s and/or RT2s ≥ 1 deep in the maxilla or mandible [25,26]; (2) full-mouth plaque score (FMPS) < 15% and full-mouth bleeding on probing (FMBOP) < 15%; (3) no systemic diseases affecting healing; (4) age ≥ 18 years.

The exclusion criteria were (1) no detectable cemento-enamel junction (CEJ); (2) gingival recessions type RT3; (3) active periodontitis; (4) cervical area with caries lesions or restorations; (5) use of medications affecting periodontal status; (6) smoking; (7) pregnancy or lactation.

Based on improvements in the percentage of root coverage and the standard deviation of measurement differences not exceeding 30% [26], the required sample size for comparing outcomes across the groups was estimated at 12 participants per arm. This calculation ensured 80% power to detect a true intergroup difference of 20 percentage points. To compensate for potential attrition, however, 20 subjects were ultimately enrolled in each treatment group.

### 2.3. Clinical Parameters

Clinical examinations were performed at baseline and 12 months after surgery by a single blinded examiner, the team’s researcher (NMC). A total of five patients not included in the trial, with more than two adjacent GR, were used to calibrate the examiner, who documented all GR recordings for each patient at 24-h intervals. The calibration was accepted when more than 90% of the measurements were repeatable within 1.0 mm of each other, and in more than 75% of the cases, the measurements were the same [27]. Using a periodontal probe (UNC probe 15 mm, Hu-Friedy, Frankfurt, Germany), all the clinical parameters were measured under local anesthesia. The records included in the trial were the following: (1) gingival recession height (GR), measured from the free gingival margin to the cemento-enamel junction (CEJ); (2) recession width (RW) at the level of the CEJ; (3) probing pocket depth (PPD), defined as the distance from the free gingival margin to the base of the gingival sulcus; (4) clinical attachment level (CAL), measured from the CEJ to the base of the sulcus; (5) keratinized tissue width (KTW), recorded as the distance between the free gingival margin and the mucogingival junction (MGJ); and (6) gingival thickness (GT), assessed 3 mm apical to the gingival margin using a size 25 ISO endodontic spreader (Poldent, Warsaw, Poland) with a silicon stopper, which was inserted perpendicularly to the gingival surface until contact with the alveolar bone or root surface was achieved.

PROMs were analyzed at the patient level to avoid inflation of sample size inherent to defect-level analysis in split-mouth designs.

### 2.4. Patient-Reported Outcomes

#### 2.4.1. Early PROMs

Early patient-reported outcomes were assessed using a structured postoperative questionnaire. Pain and swelling were evaluated using a 0–100 mm visual analogue scale (VAS), where 0 indicated no symptoms and 100 represented the worst imaginable intensity. Assessments were performed on postoperative days 1, 2, 4, 7, and 14.

In addition, the presence of postoperative symptoms, including bleeding, ecchymosis, and other complications, was recorded using dichotomous (yes/no) responses at the same time points.

#### 2.4.2. Late PROMs (12 Months)

At 12 months, patient-reported outcomes focused on esthetic perception and overall treatment experience. Esthetic outcomes were assessed using predefined items, including gingival color, gingival contour, and recession coverage, recorded as dichotomous (yes/no) responses.

Patients also evaluated overall satisfaction with the treatment outcome using a 0–100 mm VAS and reported their willingness to undergo the procedure again and to recommend the treatment to others.

All PROM questionnaires were completed at all predefined postoperative time points.

Therefore, no imputation procedures were required.

### 2.5. Professional Aesthetic Evaluation

Professional esthetic outcomes were assessed using the Root Coverage Esthetic Score (RES), a validated composite index evaluating gingival margin level, marginal tissue contour, soft tissue texture, mucogingival junction alignment, and gingival color.

One experienced periodontist (NMC) evaluated all the results at a follow-up visit 12 months after the procedure. Prior to scoring, the doctor participated in training sessions on the Root Coverage Esthetic Score (RES) using a series of pre- and post-treatment images of gingival recessions managed with different surgical approaches. No time limit was imposed for the assessments.

According to the RES system, five variables were evaluated: (1) gingival margin level (GM), (2) marginal tissue contour (MTC), (3) soft tissue texture (STT), (4) muco-gingival junction alignment (MGJ), and (5) gingival color (GC). The gingival margin level accounted for 60% of the total RES, whereas the remaining four variables accounted for 40% (10% each for MTC, STT, MGJ, and GC). GM was scored on a 0/3/6 scale, while each of the other variables was rated on a binary 0–1 scale. The maximum (optimal) esthetic score achievable for complete root coverage (CRC) was 10 points.

A score of 0 was assigned when the postoperative gingival margin was at the same level as, or more apical to, the baseline recession depth (i.e., failure of the root coverage procedure), irrespective of color match, the presence of scarring, gingival margin configuration, or MGJ position. In addition, partial or complete loss of the interproximal papilla (resulting in a “black triangle”) following treatment was also scored as 0.

*p*-values were derived from mixed-effects regression models as described in the statistical analysis section.

### 2.6. Surgical Phase

An experienced surgeon (BG) performed all periodontal procedures using the MCAT technique, which was described by Zuhr et al. [12]. In the original randomized clinical trials, allocation of treatment sites was performed using a computer-generated randomization sequence prepared by an independent statistician. Allocation was concealed in sealed opaque envelopes and revealed to the operator immediately before surgery. Right before the surgery, the operator discovered the allocation of treatment, which was concealed in sealed and opaque envelopes. The patient did not receive information on treatment allocation.

Following administration of local anesthesia with 4% articaine hydrochloride containing epinephrine (1:100,000) (Ubistesin Forte, 1.7 mL, 3-M ESPE, Saint Paul, MN, USA), the exposed root areas were instrumented with Gracey curettes (Hu-Friedy, Chicago, IL, USA). A full-thickness flap was elevated up to the mucogingival junction (MGJ), after which a split-thickness flap was created beyond the MGJ through supraperiosteal dissection. The buccal portions of the papillae were then separated from the periosteum. The free gingival graft was harvested from the palatal area. Subsequently, an SCTG was obtained by removing the outer layer extraorally. The harvested graft had a thickness of no more than 1 mm and a width of approximately 4 mm. Hemostasis at the donor site was achieved using a sponge, which was stabilized with cross-mattress non-resorbable sutures (Seralon 4/0, 18 mm, 3/8, Serag-Wiessner GmbH & Co. KG, Neila, Germany).

In the EDTA group, root surfaces were conditioned with 24% EDTA (PrefGel^®^, Straumann, Basel, Switzerland) for 2 min and rinsed with saline. In the EMD group, EDTA conditioning was followed by the application of enamel matrix derivative (Emdogain^®^, Straumann, Basel, Switzerland). In the HA group (HA; hyaDENT BG, Bioscience, Germany), root surfaces were treated with cross-linked hyaluronic acid prior to graft placement. In the saline group, the root surfaces were burnished for 2 min using cotton pellets 9 of 12 soaked in sterile saline and then rinsed with sterile saline.

In both treatment sites, a single-piece SCTG was inserted into the prepared tunnel. The graft was initially immobilized at the CEJ using resorbable sling sutures (PGA Resorba 6/0, 11 mm, 3/8; RESORBA Medical GmbH, Nürnberg, Germany). Subsequently, the coronally advanced tunnel flap was positioned to achieve complete coverage of the SCTG. Final stabilization of both the flap and graft was obtained with non-resorbable monofilament sling sutures (Seralon 6/0, 12 mm, 3/8; Serag-Wiessner GmbH & Co., 95119 Naila, Germany).

### 2.7. Post-Surgical Phase

The participants were informed to take 400 mg of ibuprofen after the procedure, another dose 6 h later, and an additional one, if required afterwards. Subsequent tablet administration was undertaken if clinically indicated. The patients were asked to avoid brushing, chewing or flossing in the perioperative area during the first 14 days. For the first 2 weeks it was recommended to gently rinse the mouth using 0.2% chlorhexidine digluconate solution twice a day for 1 min.

Two weeks after the surgical procedure, the sutures were removed, and patients were advised to use a soft toothbrush to mechanically clean the treated site using the rolling technique. Postoperative evaluations were scheduled at 1, 2, and 4 weeks, and subsequently at 3, 6, and 12 months. At each appointment, reinforcement of oral hygiene practices and professional plaque control were provided. In addition, during the postoperative phase, patient-reported outcome measures (PROMs) were systematically collected to evaluate patient-perceived morbidity and benefits of treatment, including pain and swelling/discomfort, functional limitations, and satisfaction with esthetic and functional outcomes, as described above.

### 2.8. Statistical Analyses

The statistical analyses were performed in R software version 4.2.2 (R Foundation for Statistical Computing, Vienna, Austria). Continuous variables are presented as mean ± standard deviation (SD) or median with interquartile range (IQR), as appropriate, whereas categorical variables are presented as counts and percentages.

For the descriptive and table-based comparisons, baseline characteristics presented in Table 1 were analyzed using one-way analysis of variance (ANOVA) for continuous variables and the chi-square test for categorical variables. In Table 2, VAS scores were compared among treatment groups using one-way ANOVA, whereas categorical variables, including the number and percentage of patients reporting pain or edema, were analyzed using the chi-square test. In Table 3, VAS scores were compared among treatment groups using one-way ANOVA, and categorical responses were analyzed using the chi-square test. In Table 4, within-group comparisons between baseline and 12 months were performed using paired Student’s *t*-test, whereas between-group comparisons were analyzed using one-way ANOVA. Comparisons among the four treatment groups in Table 5 were performed using one-way ANOVA.

Because of the split-mouth design, repeated measurements, and clustering of multiple recession defects within patients, the primary inferential analyses were conducted using mixed-effects regression models with patient identity included as a random intercept. Linear mixed-effects models were used for continuous clinical outcomes and VAS-based patient-reported outcome measures (PROMs), whereas generalized linear mixed-effects models with a logit link were used for binary outcomes, including complete root coverage and dichotomous PROM responses.

For repeated PROM measurements, treatment group, postoperative time point, and their interaction were included as fixed effects. Clinical outcomes were analyzed at the defect level, whereas PROMs were analyzed at the patient level. Estimated marginal means were used for pairwise comparisons, with Holm adjustment for multiple testing. Results are reported as mean differences or odds ratios with 95% confidence intervals. Model assumptions were assessed using residual diagnostics. A two-sided *p*-value < 0.05 was considered statistically significant.

## 3. Results

**Figure 1:**
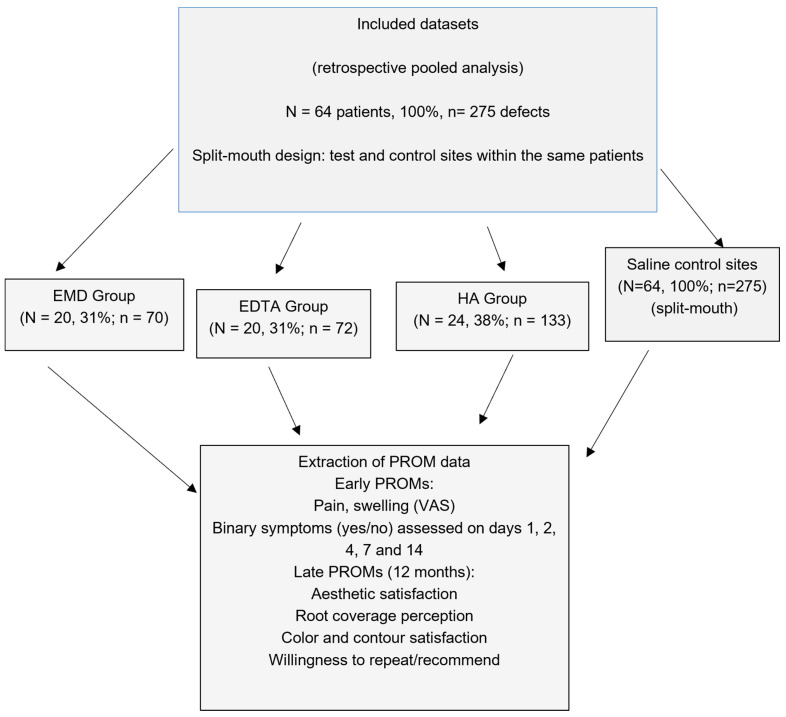
Flowchart of study design and PROM assessment. EMD: enamel matrix derivative, EDTA: ethylenediaminetetraacetic acid, HA: hyaluronic acid, N: number of patients, n: number of defects.

The flowchart of included datasets of patients and recession defects across treatment groups is presented in Figure 1.

Baseline characteristics are presented in Table 1.

### 3.1. Primary Outcome: PROMs

#### 3.1.1. Early Morbidity

Postoperative pain was common initially and decreased over time. Significant intergroup differences were observed on days 1, 2, and 7 (*p* = 0.016, 0.024, 0.033), but not on days 4 and 14. Mean VAS pain scores showed a similar pattern (*p* = 0.020, <0.0001, 0.003 vs. *p* = 0.241, 0.286). Edema was prevalent in the early phase. The proportion of patients reporting edema differed significantly on days 2, 4, and 14 (*p* = 0.011, 0.001, 0.001), but not on days 1 and 7. VAS edema scores differed significantly between groups at all time points (*p* ≤ 0.023) (Table 2).

#### 3.1.2. Long-Term Satisfaction

PROMs were highly favorable across all groups, with mean VAS scores ranging from ~76 to 90. No significant differences in overall satisfaction were observed (*p* > 0.05).

#### 3.1.3. Esthetic Perception

Satisfaction with gingival color and contour was high and comparable between groups (*p* > 0.05). Perceived recession coverage differed significantly (*p* = 0.0177) (Table 3).

#### 3.1.4. Repeat

Willingness to undergo the procedure again was high, with no intergroup differences (*p* > 0.05).

#### 3.1.5. Recommendation

Willingness to recommend treatment was high, with a significant intergroup difference (*p* = 0.0142).

### 3.2. Contextual Clinical Outcomes

A total of 64 patients with 275 gingival recession defects were included. Baseline characteristics were generally comparable between groups, although differences were observed in age and Cairo classification, with a higher proportion of RT2 defects in the HA group (Table 1).

At 12 months, all treatment modalities (EMD, EDTA, HA, and SCTG + saline) resulted in significant improvements in clinical parameters, including reductions in recession dimensions, gains in clinical attachment level, and increases in keratinized tissue width and gingival thickness. Overall, all protocols demonstrated comparable clinical effectiveness, with only minor intergroup differences, including a slightly greater CAL gain in the HA group. Although statistically significant, the magnitude of the CAL gain differences between groups was modest and should be interpreted with caution. Detailed baseline and 12-month outcomes are presented in Table 4.

### 3.3. Professional Aesthetic Evaluation

At 12 months, professional esthetic assessment showed generally high and comparable outcomes across all groups. No significant intergroup differences were observed for gingival margin and mucogingival junction scores, while differences were detected for marginal tissue contour, soft tissue texture, gingival color, and overall RES.

Overall, professional evaluations confirmed high esthetic quality across all treatment modalities and were consistent with patient-reported esthetic perception, supporting the comparability of outcomes between clinician-based and patient-based assessments (Table 5).

## 4. Discussion

The principal finding of this pooled secondary analysis is that patient-reported postoperative experience differed across adjunctive biomaterial protocols despite previously demonstrated comparable clinical outcomes. The original RCTs were previously published with clinician-centered outcomes of root coverage. The present manuscript represents a distinct secondary pooled analysis intentionally focused on PROMs and patient-perceived treatment value.

### 4.1. Early Morbidity Differences

Early healing was characterized by protocol-dependent variation in postoperative pain, swelling, and functional limitations. Although symptoms decreased over time in all groups, intergroup differences during the first postoperative week are clinically relevant, as this phase most strongly influences patient experience, treatment acceptance, and willingness to repeat surgery [28,29,30]. These differences may be partly explained by the biological properties of adjunctive agents. In particular, hyaluronic acid (HA), due to its anti-inflammatory and pro-healing effects, may contribute to improved epithelialization and reduced discomfort, consistent with previous findings [17,18,19,20,21]. However, these mechanisms remain speculative and cannot be directly inferred from the present analysis. The HA group demonstrated lower pain scores on postoperative days 2 and 4, while EDTA was associated with higher early discomfort. In contrast, EMD and EDTA primarily affect regeneration and root surface conditioning, with less direct impact on early symptom perception [16]. While such mechanisms are biologically plausible, causal inference is limited by the non-contemporaneous design [31].

### 4.2. PROMs vs. Professional Esthetics Divergence

At 12 months, clinical outcomes were comparable across all protocols, confirming that adjunctive biomaterials did not substantially influence long-term clinical effectiveness. However, patient-reported esthetic outcomes differed between groups and did not fully align with professional esthetic evaluation (RES/MTC). This divergence indicates that clinician-based indices prioritize predefined parameters of soft tissue integration, whereas patients evaluate esthetics more holistically, incorporating overall appearance, naturalness, comfort, and subjective stability [31,32,33,34]. These findings are consistent with previous reports showing that PROMs and professional assessments represent complementary but non-interchangeable dimensions of treatment success [9,10,11,12,13,14,15].

### 4.3. Clinical Implications for Shared Decision-Making

The observed pattern suggest that adjunctive protocols may differentially influence dimensions of “success” within an otherwise predictable surgical framework. While long-term clinical outcomes are comparable, early morbidity and patient-perceived esthetic benefit may vary. From a clinical perspective, these differences are relevant for individualized treatment planning and shared decision-making. Incorporating PROMs into outcome assessment allows identification of differences in early postoperative morbidity that are not captured by clinical parameters alone. Discussing expected recovery trajectories and aesthetic perception may improve patient satisfaction and treatment acceptance [31,35].

### 4.4. Limitations and Future Directions

The present findings should be interpreted with caution. The study pooled data from three split-mouth randomized controlled trials conducted at different times. Although the intra-patient saline control design preserved strong internal validity within each original dataset, the non-contemporaneous nature of the cohorts may introduce bias related to temporal factors, including operator experience, refinement of surgical techniques, and changes in perioperative management [36]. Importantly, the present analysis was not originally powered for pooled PROM comparisons. The relatively small sample size may reduce the statistical power to detect intergroup differences, particularly in PROM outcomes. The present analysis focused primarily on patient-reported outcomes, while clinical outcomes were included as contextual reference parameters.

Baseline differences, including age distribution and recession characteristics, may have further influenced both healing dynamics and patient perception. Differences in age distribution between groups may have influenced healing response and patient-reported outcomes. In addition, the use of study-specific PROM instruments and VASs limits external validity and comparability, while susceptibility to recall bias, response shift, and incomplete datasets with potential non-random missingness may introduce additional bias [36,37]. These interpretations remain hypothesis-generating.

Future studies should include adequately powered, prospective, randomized designs with contemporaneous cohorts, standardized PROM instruments, and statistical approaches that account for the clustering inherent to split-mouth designs to validate the observed differences and clarify their clinical relevance. PROM instruments used in this study were study-specific and not externally validated, which may limit comparability with other studies.

## 5. Conclusions

Within the limitations of this retrospective pooled analysis, MCAT combined with SCTG provided predictable root coverage and stable 12-month clinical outcomes in the treatment of multiple RT1 and RT2 gingival recessions, regardless of the adjunctive protocol used. Adjunctive biomaterials did not substantially influence overall clinical effectiveness. In contrast, patient-reported outcome measures (PROMs) revealed differences in early postoperative morbidity, particularly in pain and swelling during the first postoperative week, indicating that patient experience may vary despite comparable clinical outcomes. However, these differences were transient and did not translate into clinically meaningful long-term benefits. Overall, patient satisfaction, esthetic perception, and treatment acceptance were high across all groups. These findings support the integration of PROMs into clinical decision-making, allowing clinicians to better balance biological effectiveness with patient-centered outcomes when selecting among otherwise comparable treatment approaches.

## Figures and Tables

**Table 1:** Characteristics for the study groups.

Variables	EMD ^1^ (N ^4^ = 20, n ^5^ = 70)	EDTA ^2^ (N = 20, n = 72)	HA ^3^ (N = 24, n = 133)	Saline (N = 64, n = 275)	*p*
Sex (n)					0.187
Women	11	10	19	40	
Men	9	10	5	24	
Age (mean, SD ^6^)	28.47 (4.45)	29.02 (4.31)	32.54 (6.67)	30.01 (5.43)	<0.001
Tooth type (n)					0.373
Incisors	15	16	42	73	
Canines	16	17	23	56	
Premolars	33	32	45	110	
Molars	6	7	23	36	
Type o GR ^7^ according to Cairo (n, %)					<0.001
RT1 ^8^	47(67.14%)	49(68.06%)	59 (44%)	155 (59.73%)	
RT2	23(32.86%)	23(31.94%)	74 (56%)	120(40.27%)	

^1^ EMD: enamel matrix derivative, ^2^ EDTA: ethylenediaminetetraacetic acid, ^3^ HA: hyaluronic acid, ^4^ N: number of patients, ^5^ n: number of defects, ^6^ SD: standard deviation, ^7^ GR: gingival recession, ^8^ RT: recession type. Statistical method: one-way ANOVA for continuous variables; the chi-square test for categorical variables.

**Table 2:** *p*-analysis between groups (EDTA, EMD, HA, Saline).

	*p* 1st Day	*p* 2nd Day	*p* 4th Day	*p* 7th Day	*p* 14th Day
Pain—N ^1^ answering “yes” (%)	0.016 *	0.024 *	0.662	0.033 *	0.117
Pain—VAS ^2^ mean (SD ^3^)	0.020 *	<0.0001 *	0.241	0.003 *	0.286
Edema—N answering “yes” (%)	0.334	0.011 *	0.001 *	0.091	0.001 *
Edema—VAS mean (SD)	0.023 *	<0.0001 *	<0.0001 *	0.004 *	0.008 *

^1^ N number of patients, ^2^ VAS Visual Analogue Scale, ^3^ SD standard deviation, * statistically significant (*p* ≤ 0.05). Statistical method: ANOVA for VAS scores; the chi-square test for “yes”.

**Table 3:** Results of the patient questionnaire evaluating esthetics and overall satisfaction.

Question	EMD ^1^ (n ^5^ = 70)		EDTA ^2^ (n = 72)		HA ^3^ (n = 133)		Saline (n = 275)		*p*
	N ^4^ Answering “yes” (%)	VAS ^6^ Mean (SD ^7^)	N Answering “yes” (%)	VAS Mean (SD)	N Answering “yes” (%)	VAS Mean (SD)	N Answering “yes” (%)	VAS Mean (SD)	
Gingival color		81.5 (13.03)	20 (100%)	83.23 (13.35)	23 (95%)	79.8 (11.52)	84 (99%)	83.18 (11.90)	0.0796
Gingival contour		80.2 (14.89)	19 (95%)	81.89 (14.90)	24 (100%)	82.1 (13.36)	83 (98%)	83.04 (13.22)	0.2606
Recession coverage		75.8 (15.04)	19 (95%)	75.01 (19.57)	24 (100%)	82.9 (10.85)	83 (98%)	80.41 (14.63)	0.0177 *
“How satisfied are you with the results of the surgery?”	19 (95%)	83.0 (12.53)	18 (90%)	78.89 (23.11)	24 (100%)	87.4 (8.87)	82 (96%)	84.29 (14.42)	0.1812
“Would you decide again to go for the treatment performed?”	19 (95%)	84.2 (15.02)	18 (90%)	81.33 (16.61)	24 (100%)	86.9 (9.46)	82 (96%)	84.78 (13.40)	0.5395
“Would you recommend the treatment to another person?”	18 (90%)	80.8 (17.96)	18 (90%)	77.49 (16.74)	24 (100%)	87.02 (7.81)	81 (95%)	84.00 (13.90)	0.0142 *

^1^ EMD: enamel matrix derivative, ^2^ EDTA: ethylenediaminetetraacetic acid, ^3^ HA: hyaluronic acid, ^4^ N: number of patients, ^5^ n: number of defects, ^6^ VAS: Visual Analog Scale, ^7^ SD: standard deviation, * statistically significant (*p* ≤ 0.05). Statistical method: ANOVA for VAS scores; the chi-square test for “yes”.

**Table 4:** Clinical parameters (mean and standard deviation) at baseline and 12 months after surgery.

	Baseline	12 Months	*p*
GR ^1^ EMD ^2^ (mm)	1.98 (1.11)	0.21 (0.45)	<0.0001 *
GR EDTA ^3^	1.82 (1.23)	0.26 (0.72)	<0.0001 *
GR HA ^4^	1.77 (1.13)	0.12 (0.48)	<0.0001 *
GR Saline	1.76 (1.37)	0.21 (0.42)	<0.0001 *
*p*	0.4163	0.8871	
ARC ^5^ EMD (%)		94.00 (20.12)	
ARC EDTA		89.08 (31.76)	
ARC HA		84.32 (34.46)	
ARC Saline		90.4 (25.13)	
*p*		0.8871	
CRC ^6^ EMD (%)		64 (91.43)	
CRC EDTA		65 (90.28)	
CRC HA		92.12 (28,14)	
CRC Saline		84.67 (37.67)	
*p*		0.9743	
GR red ^7^ EMD (mm)		1.78 (0.99)	
GR red EDTA		1.56 (1.18)	
GR red HA		1.65 (1.09)	
GR red Saline		1.92 (1.07)	
*p*		0.3029	
RW ^8^ EMD (mm)	2.99 (1.33)	0.56 (1.23)	<0.0001 *
RW EDTA	2.76 (1.87)	0.52 (1.26)	<0.0001 *
RW HA	3.24 (1.88)	0.35 (1.29)	<0.0001 *
RW Saline	2.55 (1.67)	0.53 (1.2)	<0.0001 *
*p*	0.4163	0.8871	
PPD ^9^ EMD (mm)	1.47 (0.52)	1.76 (0.69)	0.0204 *
PPD EDTA	1.45 (0.59)	1.66 (0.68)	0.0342 *
PPD HA	1.42 (0.54)	1.42 (0.53)	0.9982
PPD Saline	1.48 (0.51)	1.72 (0.7)	0.0198 *
*p*	0.8294	0.7661	
CAL ^10^ EMD (mm)	2.56 (1.59)	1.22 (0.67)	0.0049 *
CAL EDTA	2.66 (1.65)	1.33 (0.78)	0.0104 *
CAL HA	3.08 (1.28)	0.50 (0.85)	<0.0001 *
CAL Saline	2.44 (1.68)	1.24 (0.82)	0.0183 *
*p*	0.3195	0.4178	
CAL gain EMD (mm)		2.13 (1.12)	
CAL gain EDTA		1.45 (1.10)	
CAL gain HA		2.58 (1.54)	
CAL gain Saline		1.63 (1.38)	
*p*		0.0183	
KTW ^11^ EMD (mm)	2.75 (1.33)	3.51 (1.31)	<0.0001 *
KTW EDTA	3.01 (1.32)	3.67 (1.02)	0.0018 *
KTW HA	2.80 (1.38)	3.57 (1.49)	0.2092
KTW Saline	2.85 (1.33)	3.82 (1.01)	0.0119 *
*p*	0.4107	0.3274	
KTW gain EMD (mm)		0.76 (0.99)	
KTW gain EDTA		0.79 (1.01)	
KTW gain HA		0.68 (1.40)	
KTW gain Saline		0.91 (1.05)	
*p*		0.0124	
GT ^12^ EMD (mm)	1.16 (0.34)	2.05 (0.62)	<0.0001 *
GT EDTA	1.33 (0.47)	1.93 (0.63)	<0.0001 *
GT HA	1.68 (0.72)	2.54 (0.74)	0.0351 *
GT Saline	1.28 (0.34)	2.21 (0.63)	<0.0001 *
*p*	0.1689	0.0276	
GT gain EMD (mm)		0.66 (0.55)	
GT gain EDTA		0.63 (0.57)	
GT gain HA		0.81 (0.79)	
GT gain Saline		0.73 (0.61)	
*p*		0.0458	

^1^ GR: gingival recession height, ^2^ EMD: enamel matrix derivative, ^3^ EDTA: ethylenediaminetetraacetic acid, ^4^ HA: hyaluronic acid, ^5^ ARC: average root coverage, ^6^ CRC: complete root coverage, ^7^ GR red: gingival recession reduction, ^8^ RW: gingival recession width, ^9^ PPD: probing pocket depth, ^10^ CAL: clinical attachment level, ^11^ KTW: keratinized tissue width, ^12^ GT: gingival thickness, * statistically significant (*p* ≤ 0.05). Statistical method: paired Student’s *t*-test for baseline and 12 months; ANOVA for comparisons between-group.

**Table 5:** Evaluation of esthetic outcomes after 12 months—mean (standard deviation).

	GM ^4^	MTC ^5^	STT ^6^	MGJ ^7^	GC ^8^	RES ^9^
EMD ^1^	5.62 (1.01)	0.98 (0.11)	0.95 (0.16)	0.98 (0.32)	1.00 (0.00)	9.65 (0.97)
EDTA ^2^	5.50 (1.07)	0.83 (0.21)	0.83 (0.21)	0.91 (0.33)	0.89 (0.30)	8.88 (1.22)
HA ^3^	5.75 (0.83)	0.90 (0.30)	0.96 (0.20)	0.92 (0.28)	0.98 (0.14)	9.51 (1.01)
Saline	5.57 (1.02)	0.85 (0.23)	0.86 (0.24)	0.90 (0.35)	0.85 (0.3)	8.93 (1.24)
*p*	0.7982	0.0143 *	0.0264 *	0.1241	0.0187 *	0.0091 *

^1^ EMD: enamel matrix derivative, ^2^ EDTA: ethylenediaminetetraacetic acid, ^3^ HA: hyaluronic acid, ^4^ GM: gingival margin, ^5^ MTC: marginal tissue contour, ^6^ STT: soft tissue texture, ^7^ MGJ: mucogingival junction alignment, ^8^ GC: gingival color, ^9^ RES: Root Coverage Esthetic Score, * statistically significant (*p* ≤ 0.05). Statistical method: One-way ANOVA for comparisons among treatment groups.

## Data Availability

The raw data supporting the conclusions of this article will be made available by the corresponding author on request. Access to the data is restricted at present due to administrative and technical issues related to repository registration.

## Abbreviations

The following abbreviations are used in this manuscript:

EMD: enamel matrix derivative, EDTA: ethylenediaminetetraacetic acid, HA: hyaluronic acid, GM: gingival margin, MTC: marginal tissue contour, STT: soft tissue texture, MGJ: mucogingival junction alignment, GC: gingival color, RES: Root Coverage Esthetic Score, ARC: average root coverage, CRC: complete root coverage, GRred: gingival recession reduction, RW: gingival recession width, PPD: probing pocket depth, CAL: clinical attachment level, KTW: keratinized tissue width, GT: gingival thickness, GR: gingival recession.
